# A Diagnostic Model for Screening Diabetic Retinopathy Using the Hand-Held Electroretinogram Device RETeval

**DOI:** 10.3389/fendo.2021.632457

**Published:** 2021-04-12

**Authors:** Xiaowen Deng, Zijing Li, Peng Zeng, Jing Wang, Jiaqi Liang, Yuqing Lan

**Affiliations:** ^1^ Department of Ophthalmology, Sun Yat-sen Memorial Hospital, Sun Yat-sen University, Guangzhou, China; ^2^ Guangdong Provincial Key Laboratory of Malignant Tumor Epigenetics and Gene Regulation, Sun Yat-sen Memorial Hospital, Sun Yat-sen University, Guangzhou, China

**Keywords:** diabetic retinopathy, electroretinogram, diagnostic model, risk factor, decision tree

## Abstract

**Purpose:**

To construct a proper model to screen for diabetic retinopathy (DR) with the RETeval.

**Method:**

This was a cross-sectional study. Two hundred thirty-two diabetic patients and seventy controls were recruited. The DR risk assessment protocol was performed to obtain subjects’ DR risk score using the RETeval. Afterwards, the receiver operating characteristic (ROC) curve was used to determine the best cutoff for diagnosing DR. Random forest and decision tree models were constructed.

**Results:**

With increasing DR severity, the DR score gradually increased. When the DR score was used to diagnose DR, the ROC curve had an area under the curve of 0.881 (95% confidence interval: 0.836-0.927, P < 0.001), with a best cutoff value of 22.95, a sensitivity of 74.3% (95 CI: 66.0%~82.6%), and a specificity of 90.6% (95 CI: 83.7% ~94.8%). The top four risk factors selected by the random forest were used to construct the decision tree for diagnosing DR, which had a sensitivity of 93.3% (95% CI: 86.3%~97.0%) and a specificity of 80.3% (95% CI: 72.1% ~86.6%).

**Conclusions:**

The DR risk assessment protocol combined with the decision tree model was innovatively used to evaluate the risk of DR, improving the sensitivity of diagnosis, which makes this method more suitable than the current protocol for DR screening.

## Introduction

Diabetic retinopathy (DR) is a serious chronic complications of diabetes mellitus (DM), and it is the main cause of sight loss among the working population worldwide (1). With the continuously increasing prevalence of diabetes in recent years (2), early diagnosis and treatment of DR has become increasingly important. China has the largest population of diabetes patients in the world (3), and the prevalence of DR in rural areas with insufficient medical resources is higher than that in urban areas (4). At present, the diagnostic methods of DR mainly rely on professional ophthalmologists. In primary medical institutions, such as community hospitals, where there is a lack of professional ophthalmologists and examination equipment, it is difficult to conduct professional eye examinations, which also makes clinical follow-up more difficult.

In the past few years, there has been increasing evidence that neurodegenerative changes in diabetic patients occur during preclinical DR (before microvascular changes occur) (5, 6). However, traditional flash electroretinogram (FERG) devices and multifocal electroretinogram (mfERG) devices are time consuming to use. In addition, traditional electroretinogram devices require pupil dilation, the use of invasive corneal electrodes, and professional analyses (7, 8), which greatly reduce the efficiency of the device. The advent of the RETeval, a hand-held ERG device, has made it much easier to make general judgments about retinal function in the community and to perform initial DR screening. The RETeval (LKC Tech. Inc., Gaithersburg, MD, USA) is a small, handheld FERG-recording device that uses special skin electrodes to capture ERGs. The device can perform a FERG test without pupil dilation noninvasively and quickly. Traditional FERG and mfERG reports have no intuitive judgment criteria and need professional interpretation. The DR risk assessment protocol of the device calculates the implicit time, amplitude, and pupillary response of flicker ERGs at 30 Hz to obtain a DR risk score. Compared to traditional ERG examinations, DR screening, even by nonprofessionals in primary care settings, can reduce subjective errors and make it more feasible. This device has been effective in studies of ERG in diabetic retinopathy (9–11) and has good reproducibility (12), but it has a high misdiagnosis rate in early DR screening when using the device directly. In addition, there may be differences among different races. The purposes of this study were to find the appropriate diagnostic threshold in South Chinese diabetic patients and to establish a simple and effective screening model, so as to popularize the screening for DRin the community. The risk factors from its use were also assessed.

## Methods

### Subjects

This was a cross-sectional observational study. The study adhered to the tenets of the Declaration of Helsinki and was approved by the research ethics committee of Sun Yat-sen Memorial Hospital, Sun Yat-sen University. Two hundred thirty-two patients with type 2 diabetes mellitus (T2DM) recruited from the DM center between March 2019 and January 2020 and seventy healthy controls were included in the study. All were ethnically Chinese, mostly from southern China. DR stages were determined according to the criteria published by the ADA in 2017 (13). A randomly selected eye was included from each healthy, no-DR (NDR) control patient and each DR patient with the same DR stage in both eyes, while the worst eye was selected if the patient had uneven DR severity in the two eyes. Vision-threatening diabetic retinopathy (VTDR) was defined as severe nonproliferative diabetic retinopathy (NPDR), proliferative diabetic retinopathy (PDR), or clinically significant macular edema (CSME) with any stage of DR. The diagnosis of CSME was based on slit lamp fundus examination, fundus photography, and optical coherence tomography (OCT) examination and was defined as: (1) retinal thickening within 500 μm of the macular fovea, (2) macular fovea showing hard exudation within 500 μm and related to the thickening of the adjacent retina, or (3) retinal thickening in one or more places ≥1 papilla diameter and distance from macular fovea <1 papilla diameter. The exclusion criteria were as follows: (1) eye diseases such as glaucoma, uveitis, spherical equivalent >6 diopters, etc.; (2) ocular trauma or ocular surgical history (including retinal photocoagulation and intravitreal injection); (3) craniocerebral trauma or surgeries and ischemic diseases; (4) acute kidney disease or malignant hypertension; (5) photosensitive epilepsy; and (6) opaque refractive media or an ungradable fundus.

All subjects underwent a detailed ocular examination, including LogMAR best-corrected visual acuity (BCVA), noncontact tonometer intraocular pressure (IOP) (NIDEK, Inc., Aichi, Japan), axial lengths (by the IOLmaster, Zeiss, Inc., Jena, Germany), fundus photography (Canon, Inc., Tokyo, Japan), optical coherence tomography (OCT), and mydriatic slit-lamp fundus examination. The OCT examination was performed with the RTVue XR Avanti device (Optovue, Inc., Fremont, CA, USA) in 6.0×6.0 mm B-Scan mode after mydriasis. The stage of DR was confirmed by two experienced ophthalmologists according to the results of slit-lamp fundus examination, color fundus photographs, OCT, and fundus fluorescein angiography (FFA, Microclear, Inc., Suzhou, China) in suspected PDR patients. FFA images were collected as 9-field 60° fundus photographs after mydriasis. Age, sex, DM duration, glycosylated hemoglobin (HbA1c) levels, and body mass index (BMI) were collected. Moreover, the presence of systemic diseases, including high blood pressure(HBP), impaired renal function (IRF), dyslipidemia, and diabetic complications, including diabetic peripheral neuropathy (DPN), diabetic peripheral vasculopathy (DPV), and diabetic foot, was also recorded. IRF was defined as: (1) a history of chronic kidney diseases or diabetic nephropathy, (2) estimated glomerular filtration rate (eGFR) <60 ml/min/1.73 m2, (3) urinary albumin:creatinine ratio >30 mg/g for more than 3 months, and (4) need for a renal biopsy in suspected patients. eGFR was calculated from serum creatinine according to the Xiangya equation (14). All the above indexes were classified as dichotomous variables (Yes/No) based on the presence or absence of diseases or dysfunctions. A diagnosis of hypertension (>130/80 mmHg) was made according to associated guidelines updated in 2017 by the American College of Cardiology/American Heart Association (ACC/AHA) (15).

### FERG Examination

The FERG examination was performed by the RETeval. Special skin electrodes of the RETeval device and the nondilated pupil mode of the DR risk assessment protocol were used for examination. The DR risk assessment protocol of the device was provided by the manufacturer. The original calculation method was established based on multiperson research by Maa et al. to screen VTDR (9). A 30-Hz flicker ERG, as set in the electrophysiological standard by the International Society for Clinical Electrophysiology of Vision (ISCEV) (8), was used to observe the cone cell response. The time delay (implicit time) between the stimulus and the peak electrical response, as well as the peak-to-peak amplitude of the electrical response, was recorded after the scintillation photostimulus was administered. The device provides fixed retinal illuminating (Td-s) stimulation by adjusting brightness (cd-s/m2); therefore, FERG can be recorded without dilated pupils to compensate for changes in pupil area (mm^2^) (16). A flashing white-light stimulus is made up of brief (< 5 ms) flashes from red, green, and blue LEDs at a frequency of 28.3 Hz with a background light of 0 Td-s. After it recorded the implicit time and amplitude of 16 Td-s and 32 Td-s flashes, as well as the pupil area ratio between 4 Td-s and 32 Td-s flashes, it generated a report including the parameters above and a DR risk assessment score (called DR score) calculated from them for each eye. The default normal value range is 7-19.9, and a DR score greater than or equal to 20 suggests a high risk of VTDR.

### Statistical Analysis

The comparative analysis of data was done with SPSS 25.0 (SPSS Inc. Chicago, IL, USA), a commercial statistical program. One-way ANOVA was used to analyze the numerical variables among the groups, and Bonferroni’s *post hoc* analysis was applied to evaluate statistical significance. Categorical variables were analyzed by the chi-square test. In all diabetic patients (DM with no DR, NPDR, or PDR), the receiver operating characteristic (ROC) curve to screen DR or VTDR was constructed by using the DR score and the stages of DR, and the area under the curve (AUC) was determined. The sensitivity and specificity were obtained according to the ROC curve, and the optimal diagnostic cutoff point was obtained by using the maximum value of the Youden index (YI= sensitivity + specificity -1). The significance levels of all the above statistical tests were set at 0.05.

R software (http://www.r-project.org) was used to analyze the risk factors and construct the DR screening model. The randomforest package was applied to analyze the risk factors and build the random forest. The mean decrease Gini (MDG) obtained by randomforest indicated the correlation between various factors and DR, in which a larger MDG of the factor meant a greater influence on DR. The out-of-bag (OOB) error estimate, which was computed by the OBB classifier on the training set, was as accurate as the error rate obtained by using the test set with the same size as the training set and let us avoid creating a separate set of tests. The Rpart package of R software was applied to obtain a decision tree. A decision tree is a nonlinear discriminant method that can divide the sample into subgroups. In the current model, the target variable was whether DR or VTDR was present. Starting at the root, the data were divided into two groups at each node according to whether the most correlated factors met the criteria. The process was then repeated for each node until all subjects were assigned to either a high-risk or a low-risk group. The confidence intervals of the ROC curves and decision trees were calculated by the efficient-score method (17).

## Result

### Subject Characteristics

Two hundred thirty-two eyes of 232 T2DM subjects (127 NDR and 105 DR) and seventy eyes of 70 matched healthy controls were included in this study. There were no significant differences in age or sex among the three groups. Compared with the NDR group, the patients in the DR group had a longer course of diabetes, a higher level of glycosylated hemoglobin, and a higher prevalence of HBP, IRF, diabetic foot, and DPN (P < 0.05), while there was no significant difference between the two groups in terms of dyslipidemia and DPV. BCVA showed no significant difference between the healthy control group and the NDR group, while visual acuity decreased significantly in the DR group compared with the controls and the NDR group (P < 0.001). There was no significant difference in the ocular axis or IOP among the three groups. Details are given in **Table 1**. 

**Table 1:** Subject characteristics.

Group	Controls	NDR	DR	P VALUE		
	(n=70)	(n=127)	(n=105)	Controls vs NDR	Controls vs DR	NDR vs DR
Genger (M/F)	36/34	77/50	67/38		*χ^2 =^*2.770*,P* =0.250	
Age (Year)	54.24 ± 9.55	55.73 ± 12.97	57.37 ± 8.39	1.0	0.186	0.739
BMI (kg/m^2^)	NA	24.79 ± 3.49	23.96 ± 3.17	NA	NA	0.06
HbAlc%	NA	8.40 ± 2.06	9.69 ± 2.44	NA	NA	<0.001*
Duration of DM (Year)	NA	7.36 ± 6.78	11.32 ± 5.84	NA	NA	<0.001*
HBP (YES/NO)	NA	64/63	68/37	NA	* χ^2 =^*4.839*,P*=0.028*	
IRF (YES/NO)	NA	25/102	51/54	NA	* χ^2 =^*21.774*,P*<0.001*	
Diabetic Foot (YES/NO)	NA	2/125	12/93	NA	* χ^2 =^*9.842*,P*=0.002*	
DPV (YES/NO)	NA	55/72	49/56	NA	*χ^2 =^*0.262*,P*=0.609	
DPN (YES/NO)	NA	58/69	74/31	NA	* χ^2 =^*14.423*,P*<0.001*	
Dyslipidemia (YES/NO)	NA	62/65	54/51	NA	*χ^2 =^*0.157*,P*=0.692	
Axial length (mm)	23.28 ± 1.00	23.70 ± 1.23	23.31 ± 0.76	0.231	1.0	0.101
IOP (mmHg)	14.97 ± 2.36	15.01 ± 2.58	16.00 ± 3.81	1.0	0.315	0.193
BCVA (Logmar)	-0.03 ± 0.11	-0.03 ± 0.06	0.31 ± 0.39	1.0	<0.001*	<0.001*

NDR, no diabetic retinopathy; DR, diabetic retinopathy; M, male; F, female; BMI, body mass index; HBP, high blood pressure; IRF, impaired renal function; DPV, diabetic peripheral vasculopathy; DPN, diabetic peripheral neuropathy; IOP, intraocular pressure; BCVA, best-corrected visual acuity. *Statistically significant.

### FERG Findings

**Figure 1:**
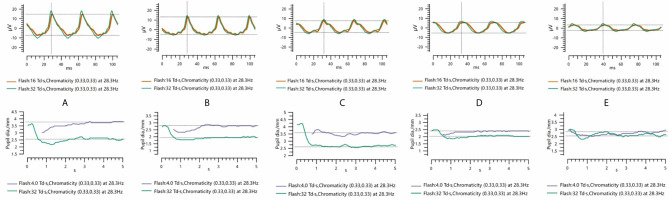
The representative 30Hz flicker ERG performances and pupil diameter ratios of control **(A)**, no DR **(B)**, mild NPDR **(C)**, moderate-severe NPDR **(D)**, and PDR **(E)**.

**Figure 2:**
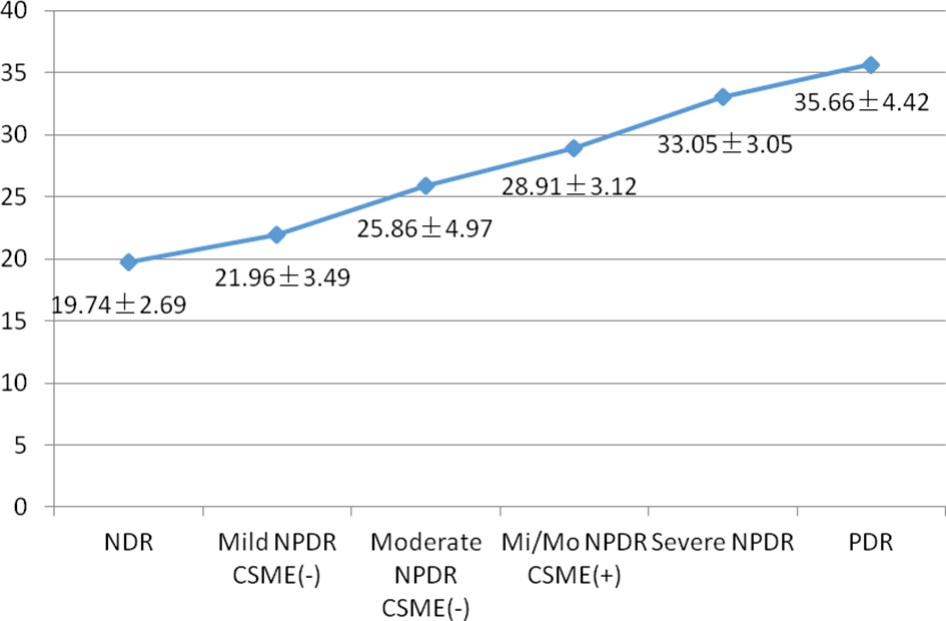
Mean DR score in various stages of DR. NDR, no diabetic retinopathy; NPDR, non-proliferative diabetic retinopthy; Mi/Mo NPDR, mild or moderate non-proliferative diabetic retinopthy; PDR, proliferative diabetic retinopthy.

The features of FERG at each stage of DR are shown in **Figure 1**. In this study, as DR severity increased, the amplitude of ERG gradually decreased, the implicit time gradually extended, and the pupillary response gradually deteriorated. The details and comparison of the parameters in the DR assessment protocol are shown in **Table 2**. The DR score increased successively from the healthy control group to the NDR group to the DR group (18.35 ± 2.56 in the healthy control group, 19.74 ± 2.69 in the NDR group and 28.37 ± 6.43 in the DR group). The implicit time of 16Td-s and 32Td-s grew successively longer from the healthy control group to the NDR group to the DR group. The amplitudes of 16Td-s and 32Td-s and the pupillary area were decreased from the healthy control group to the NDR group to the DR group. Between the healthy control group and NDR group, only the amplitudes of 16Td-s and 32Td-s flashes were significantly different (P=0.001), while there was no significant difference in DR score, latency, or pupil response between the two groups (P > 0.05). All values showed statistically significant differences between the healthy control group and DR group and between the NDR and DR groups (P < 0.05). As shown in **Figure 2**, when all DM patients were divided into no-DR, mild NPDR, moderate NPDR, mild/moderate NPDR with CSME, severe NPDR, and PDR groups, the DR score tended to increase with the progression of DR.

**Table 2:** Parameters of the DR risk assessment protocol.

Group	Controls	NDR	DR	P VALUE		
	(n=70)	(n=127)	(n=105)	Controls vs NDR	Controls vs DR	NDR vs DR
DR score	18.35 ± 2.56	19.74 ± 2.69	28.37 ± 6.43	0.099	<0.001*	<0.001*
16 Td-s implicit time(ms)	28.71 ± 1.46	29.66 ± 2.19	35.28 ± 4.73	0.305	<0.001*	<0.001*
16 Td-s amplitude(μV)	19.81 ± 4.96	16.04 ± 6.61	10.39 ± 5.44	0.001*	<0.001*	<0.001*
32 Td-s implicit time(ms)	27.64 ± 1.22	28.53 ± 1.83	34.37 ± 4.90	0.359	<0.001*	<0.001*
32 Td-s amplitude(μV)	23.56 ± 5.46	19.08 ± 7.57	12.55 ± 6.10	0.001*	<0.001*	0.001*
Pupil area ratio	2.01 ± 0.39	1.91 ± 0.48	1.53 ± 0.29	0.440	<0.001*	<0.001*

NDR, no diabetic retinopathy; DR, diabetic retinopathy. *Statistically significant.

### ROC Curves

**Figure 3:**
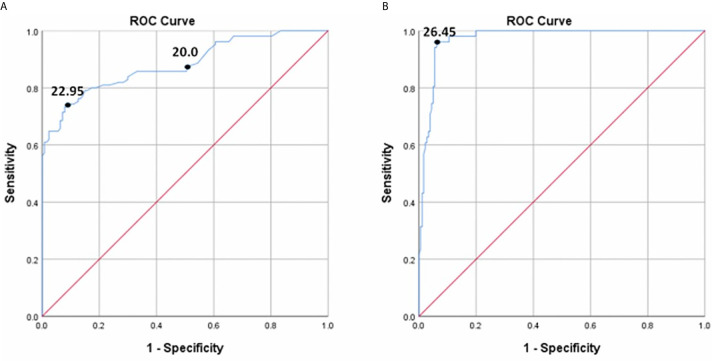
The ROC curve for DR score to detect DR **(A)** and VTDR **(B)**.

In all diabetic patients (NDR and DR), the ROC curves for detecting DR and VTDR using the DR score are shown in **Figure 3**. To screen for DR, the area under the ROC curve (AUC) was 0.881 (95% confidence interval (CI) 0.836-0.927, *P* < 0.001), and the optimal cutoff value was 22.95, with a sensitivity of 74.3% (95 CI: 66.0%~82.6%), a specificity of 90.6% (95 CI: 83.7% ~94.8%), and a YI of 0.648. If the default threshold of 20.0 of the DR risk assessment protocol were used to diagnose DR, the sensitivity would be 87.6% (95 CI: 79.4% ~93.0%), with a specificity of 48.8% (95 CI: 39.9% ~57.8%), and a YI of 0.364. When detecting VTDR using the DR score, the AUC was 0.972 (95% CI: 0.954-0.991, P < 0.001) with the best threshold of 26.45, a sensitivity of 95.7% (95% CI: 84.3% ~99.3%), a specificity of 93.5% (95% CI: 88.7% ~96.5%), and a YI of 0.894.

### Random Forest

**Figure 4:**
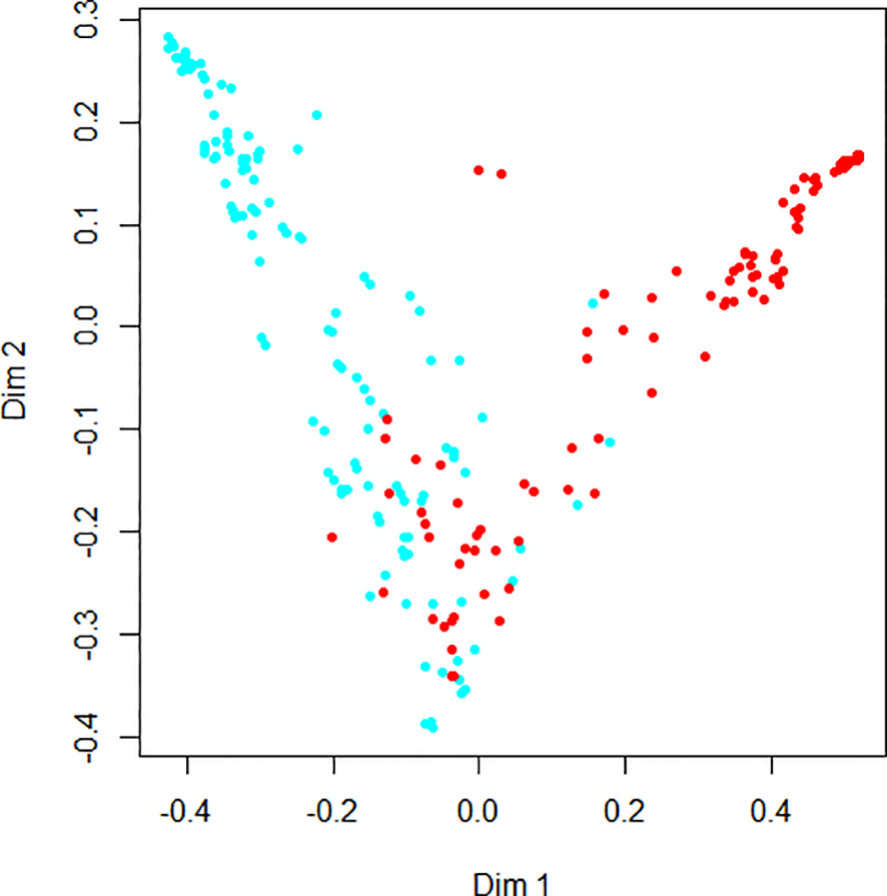
The random forest map for diabetic retinopathy detection.

**Figure 5:**
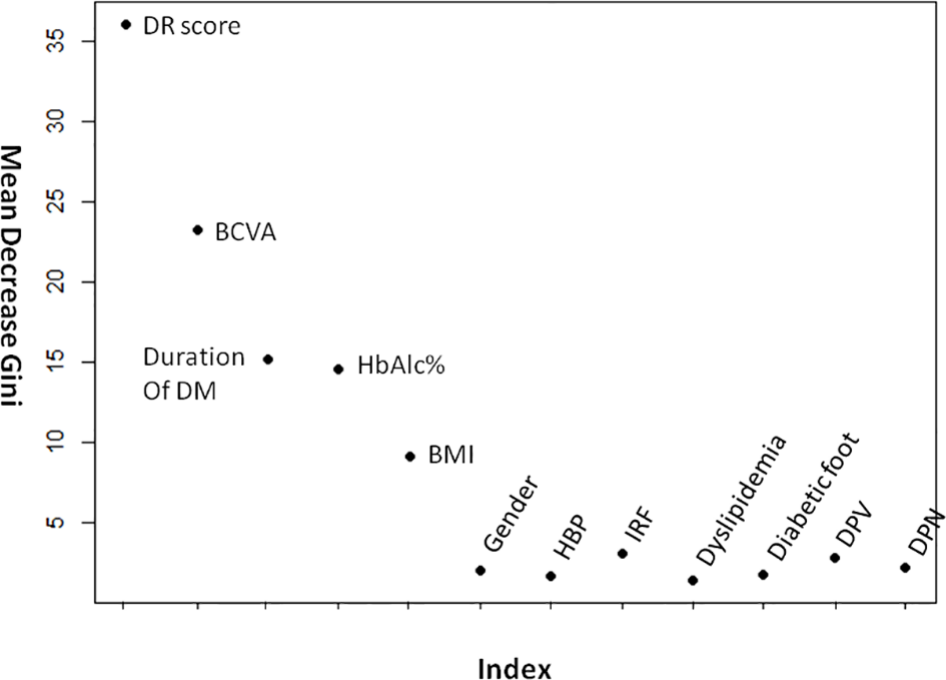
The mean decrease gini values of DR- related factors. BCVA, best-corrected visual acuity; BMI, body mass index; HBP, high blood pressure; IRF, impaired renal function; DPV, diabetic peripheral vasculopathy; DPN, diabetic peripheral neuropathy.

**Figure 4** demonstrates the random forest map based on the presence of DR and the DR score, as well as the related risk factors mentioned above. Red dots represent DR subjects, blue dots represent NDR subjects, and the OOB estimate of the error rate is 4.74%. **Figure 5** shows the MDG values of the factors. The top several were DR score (36.05), BCVA (23.21), duration of DM (15.14), HbA1c (14.58), BMI (9.13), and IRF (3.06), while the MDG values of the other indexes (gender, HBP, dyslipidemia, diabetic foot, DPV, and DPN) were less than 3.

### Decision Trees

**Figure 6:**
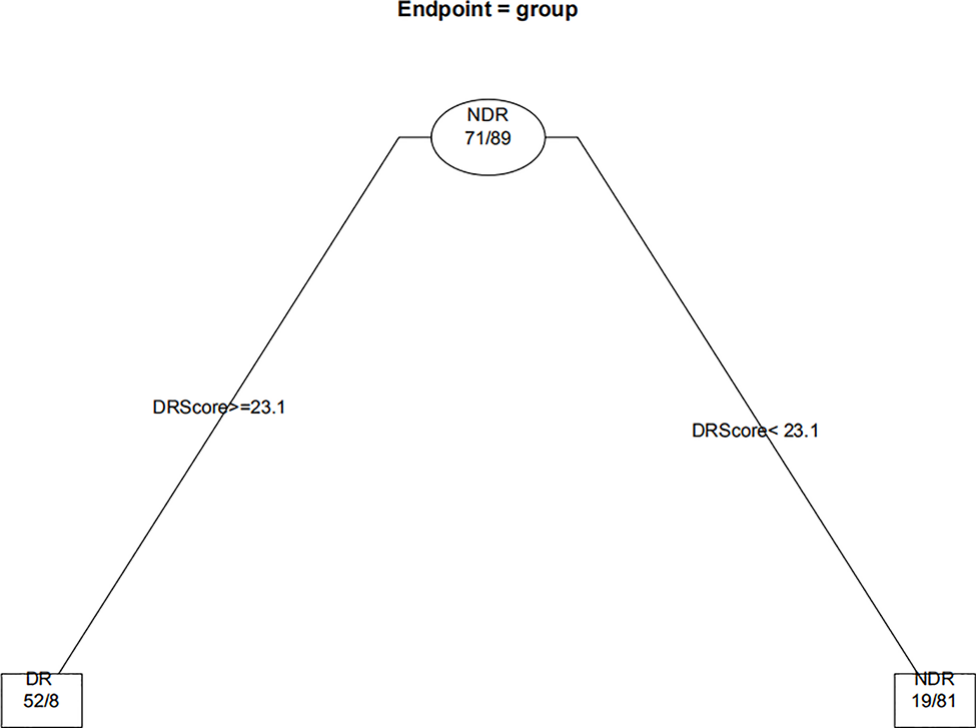
Decision tree for detecting DR using only DR score.

**Figure 7:**
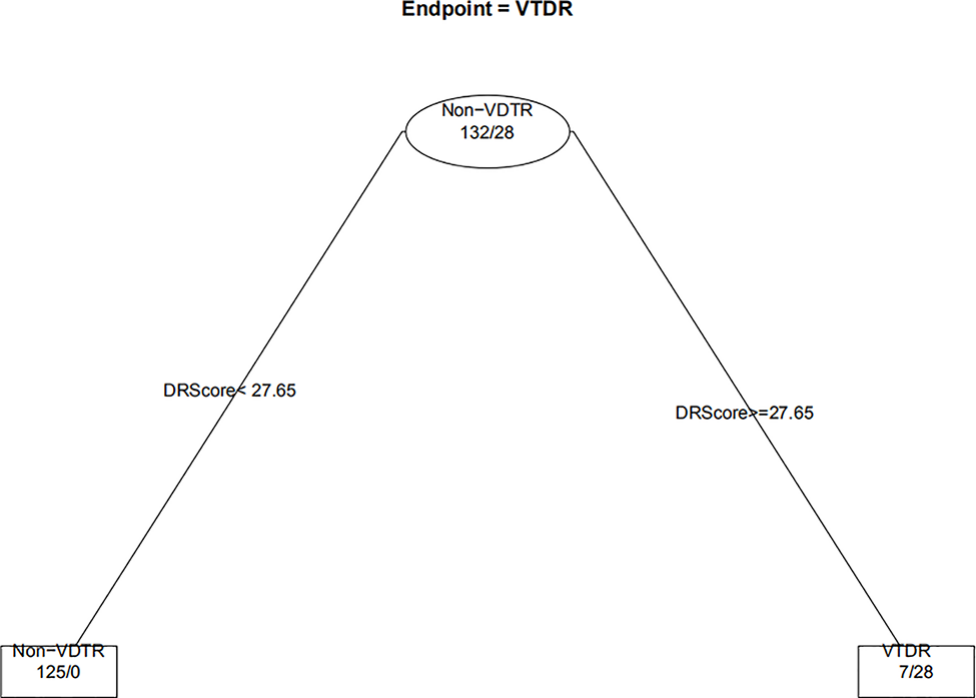
Decision tree for detecting VTDR using only DR score.

When the decision trees were constructed, the 232 eyes of the DM patients were divided into 160 eyes for the training set (approximately 7/10) and 72 eyes for the test set (approximately 3/10). **Figures 6** and **7** show the decision trees of DR and VTDR, respectively, decided by only the DR score using the training set. In the simple model for DR, it had a sensitivity of 70.6% (95% CI:52.3%~84.2%) in the training set and 72.4% (95% CI:62.6%~80.4%) in the test set, and a specificity of 92.1% (95% CI:77.5%~97.9%) in the training set and 91.3% (95% CI:84.6%~95.4%) in the test set. For VTDR, the simple decision tree had a sensitivity of 100.0% (95% CI:85.0%~100.0%) in the training set and 100.0% (95% CI:79.1%~100.0%) in the test set, and a specificity of 94.6%(95% CI:89.0%~97.7%) in the training set and 92.5%(95% CI:80.9%~97.6%) in the test set.

**Figure 8:**
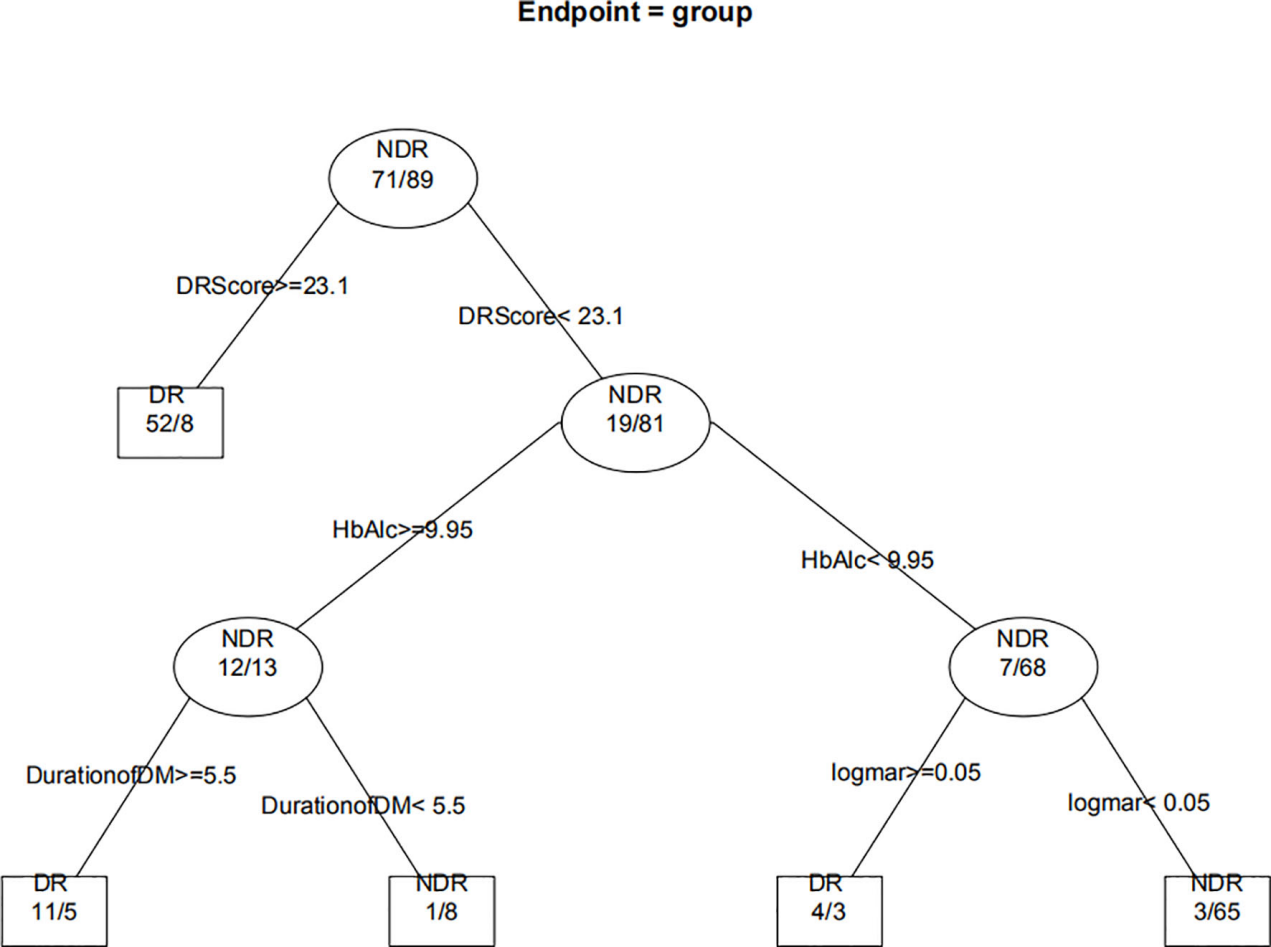
Decision tree for detecting diabetic retinopathy using DR score combined with several factors. The subsets in ellipses means the classification had not been completed, while the subsets in the boxes mean completed. Subsets in “DR” boxes mean “ high risk of DR”, while “NDR” subsets mean “low risk of DR”. The numbers in every subset mean “true DR subjects/true NDR subjects”. For example, the first box in the upper left corner means 60 subjects were considered to be at high risk for DR, of which 52 were true cases of DR and eight were actually NDR subjects. For the last box, it means 68 subjects were considered to be at low risk for DR, of which three were actually cases of DR and 65 were true NDR subjects.

**Figure 8** displays the decision rules of the factor-combined decision tree for detecting DR using the training set. The top several factors (DR score, BCVA, duration of DM, HbA1c%, BMI, and IRF) obtained from the random forest were included in the Rpart package, and DR score, BCVA, duration of DM, and HbA1c% were selected by the program to build the decision tree. **Tables 3**–**5** show the results of DR screening in the training set, the test set, and the summation, respectively, of which display the comparison of the factors-combined model and the DR score-only model. Adding up the results, the decision tree with risk factors to detect DR had a sensitivity of 93.3% (95% CI: 86.3%~97.0%) and a specificity of 80.3% (95% CI: 72.1% ~86.6%), while the DR-score-only model had a sensitivity of 72.4% (95% CI: 62.6%~80.4%) and a specificity of 91.3% (95% CI: 84.6%~95.4%).

**Table 3:** The classification results of the train sets for screening DR.

	True value	Predicted value		sensitivity	specificity
		case	control		
Factor-combined model	case	67	4	94.4% (95% CI:85.5%~98.2%)	82.0% (95% CI:72.1%~89.1%)
	control	16	73		
DR score only model	case	52	19	73.2% (95% CI:61.2%~82.7%)	91.0% (95% CI:82.6%~95.8%)
	control	8	81		

**Table 4:** The classification results of the test sets for screening DR.

	True value	Predicted value		sensitivity	specificity
		case	control		
Factor-combined model	case	31	3	91.2% (95% CI:75.2%~97.7%)	76.3% (95% CI:59.34%~88.0%)
	control	9	29		
DR score only model	case	24	10	70.6% (95% CI:52.3%~84.2%)	92.1% (95% CI:77.5%~97.9%)
	control	3	35		

**Table 5:** The classification results of all diabetes subjects for screening DR.

	True value	Predicted value		sensitivity	specificity
		case	control		
Factor-combined model	case	98	7	93.3% (95% CI:86.3%~97.0%)	80.3% (95% CI:72. 1%~86.6%)
	control	25	102		
DR score only model	case	76	29	72.4% (95% CI:62.6%~80.4%)	91.3%( 95% CI:84.6%~95.4%)
	control	11	116		

## Discussion

In this study, with the progression of DR, DR scores gradually increased, with longer implicit times and decreased amplitudes of 30-Hz flicker ERG, as well as worse pupil responses. Previous studies on DR assessment protocols have shown the same trend (9, 11, 18). Changes in flicker ERG at 30 Hz were associated with the severity of DR. When DR progresses with increased retinal ischemia, apoptosis of retinal cells, especially ganglion cells (19), leads to impaired retinal function, which induces a prolonged implicit time and a decreased amplitude (20). The speed and amplitude of pupillary contraction after light stimulation decreases with increasing DR severity, and an impaired pupillary dilatation and light reflex response in diabetes, may be due to sympathetic neuropathy or parasympathetic dysfunction (21). When the pupil is not artificially dilated, it can act as an independent indicator of the severity of DR (9).

Although the DR score showed no significant difference between the healthy control and NDR groups, we believe that the decreased amplitudes of 16Td-s and 32Td-s flicker stimuli suggested that functional impairment may have occurred before identifiable retinopathy appeared in the diabetes patients. Zeng’s research showed that NDR patients had a lower amplitude and longer implicit time than healthy people by 30-Hz flicker ERG (22), while Tyrberg’s study showed only a longer implicit time (23). In Fukuo’s studies, both the amplitude and implicit time of 8Td-s flash were not significantly different between the healthy control group and the NDR group (10), which we suspect may be related to the weaker intensity of light stimulation (24). The results of animal experiments have also varied (25, 26). The 30-Hz flicker ERG is the response of the cones (8), where the density of cone cells in the macular fovea is higher (27); therefore, only if the entire retina or the macula is involved, there is a significant change. In other words, if the macula is involved, the test becomes more sensitive and helpful to evaluate the effectiveness of treatment (28). In addition, traditional ERG examinations required dark adaptation (8) and were time consuming, while a 30-Hz flicker ERG check can be done in a few minutes.

In this study, compared with the use of the RETeval to diagnose any DR, the sensitivity and specificity for detecting VTDR were increased, which suggested that its diagnostic value in early DR is not as good as that in a more serious stage of DR. Previous studies have shown the same trend. In Fukuo’s studies, the sensitivity and specificity of the optimal cutoff point for any DR diagnosis were 0.70 and 0.81, while the sensitivity and specificity for the diagnosis of severe NPDR were 0.85 and 0.85, respectively (10). In Zeng’s research, the sensitivity and specificity for any DR were 80.2% and 81.7%, respectively, while the sensitivity and specificity for VTDR were 94.6% and 88.8%, respectively (18). Therefore, more information should be used to assess a patient’s fundus. A random forest model was introduced for the first time to analyze the DM and DR-related risk factors of patients. A random forest with a low classification error rate (OBB error estimate=4.74%) was established. The sensitivity of the decision tree model combined with the top several risk factors (DR SCORE, BCVA, duration of DM, and HbA1c%) calculated by the random forest model can also offer an improvement. In this way, we can get an overall impression of DM patients by performing RETeval and obtain some simple indexes, such as best corrected visual acuity, diabetes course, and the level of blood glucose, which are effective and useful, especially in community and clinical follow-ups. Given the 18.45%–23% prevalence of DR in Chinese diabetes patients (29, 30), we assumed there are 200 DR patients out of 1000 diabetes subjects. If we used the best cutoff of 22.95 obtained from the ROC curve of this study to screen for DR, 149 patients would be screened out, and 51 patients would be missed. At the same time, 725 of the 800 patients with no DR were considered low risk for DR, and 75 were considered high risk for DR. In conclusion, the positive predictive value and negative predictive value of DR screening using the DR score alone were 66.5% and 93.4%, respectively. Similarly, the positive predictive value and negative predictive value of VTDR screened by DR score were 78.6% and 98.8%, respectively, and the positive predictive value and negative predictive value of the decision tree model combining several risk factors were 54.2% and 98.0%, respectively. The model combining risk factors increased the number of patients who were misidentified as high risk by 83, but it also reduced the number of missed diagnoses by 38.

In the current study, the top several factors related to DR arranged according to the MDG value were, respectively, DR score (36.05), BCVA (23.21), duration of DM (15.14), HbA1c (14.58), and BMI (9.13). The MDG values of other indexes (IRF, sex, HBP, dyslipidemia, diabetic foot, DPV, and DPN) were around or less than 3. Previous studies have found that poorer blood glucose control and longer diabetes duration are strongly associated with DR (1, 31), and high blood glucose levels can lead to pericyte loss, capillary occlusion, microangioma formation, and other problems (32). With the increase in the duration of diabetes, the deterioration of retinal function might be correlated with the increase in vascular endothelial growth factor level (33). Van’s study showed that obesity was associated with retinopathy, while others found no association (34). Another study found that hypertension, dyslipidemia, vascular risk factors, diabetic peripheral neuropathy, and renal function were correlated with retinopathy (34–37), while this study found no such associations. We suspect this discrepancy was related to the selection of subjects and the sample size. In addition to the DR score, these indicators (BCVA, duration of DM, and HbA1c) were also selected to build a decision tree, suggesting that their correlation with DR may be stronger. Therefore, diabetic patients should pay close attention to the control of blood glucose and check whether there is any change in BCVA, and patients with long diabetes durations should be especially vigilant.

In China, no national DR screening system has been established, and DR screening has not been carried out in most parts of China (38). If not treated in time, DR will seriously impair vision, which often creates great familial and socioeconomic burdens (39) and eventually leads to blindness (1). The DR assessment protocol of the RETeval detects abnormalities in retinal function that come from diabetes and produces an objective DR risk score. The RETeval can be operated and read through simple training without specialized ophthalmologists. Moreover, ERG data can be documented for pretreatment and posttreatment follow-ups (40). At present, it is generally believed that, compared with fundus photography and optical coherence tomography, electrophysiological examinations in cases of affected intraocular refractive media, such as cataracts and vitreous hemorrhage, are more effective and can be used as prognostic assessments of postoperative visual acuity (41, 42). Although Miura’s study claimed that the device was affected by cataracts (43, 44), Ratanapakorn’s study showed that the differences were not statistically significant (45). In the current study, subjects with other ocular diseases that may affect ERG results were also excluded. However, these patients could also benefit from the device if they could be directed to ophthalmic specialists after examination by the device.

The current study and Mehmet’s study examined each eye to generate a separate DR risk assessment report (11). Maa and Zeng combined two-eye tests to produce a DR score (9, 15). We wanted to detect DR monocularly to set the scope, rather than to assess the overall risk of DR. Although most DR patients follow the principle of binocular congruence, and using both eyes can assess the risk of DR as a whole, we still found many DR patients; 18.1% (19/105) of DR subjects in this study had unequal severities of DR in two eyes, and seven patients had only the worst eye reach the level of VTDR, which needed further treatment soon. The use of binocular grading does not reflect each eye alone, and for patients with only one eye, it is necessary to evaluate one eye separately. The purposes of the two examination modes are different. Therefore, compared with Maa’s study (sensitivity of 83% and specificity of 78%) (9) and Zeng’s study (sensitivity of 94.6% and specificity of 88.8%) (15), ours may be more sensitive (96.1%) in detecting VTDR. Therefore, it is necessary to further optimize techniques, correct algorithms, or combine this method with other factors or devices to reduce classification mistakes.

We found that the mean DR scores in this study were higher than those of previous studies. Maa’s study was primarily in Caucasian and African subjects, with a best cutoff of 20.0 for screening VTDR (9). In Mehmet’s study, Turkish subjects were selected, and the best cutoff for screening moderate NPDR or more severe DR was 22 (11). Previous studies showed that the amplitudes of people with light-colored choroids were higher than those with dark pigmentation (46). All of our subjects were Chinese, and had dark-colored choroids, which might lead to a decrease in amplitude (47). This would be related to increased resistance associated with melanin or reduced effective illumination of the retina, thus reducing ERG amplitude (47, 48). Moreover, the worst eyes were selected, which meant lower amplitudes, prolonged implicit times, and poorer pupil responses. In addition, the higher DR scores in our study may be related to the poor blood glucose control of the subjects (mean HbA1c% of 8.40 in NDR subjects, mean HbA1c% of 9.69 in DR subjects) (31, 32), who were recruited from a DM center. Therefore, it is recommended that each examination room establish its own normal range and reference boundaries due to the differences between races, regions, and instruments (8).

### Limitations

Due to the small sample size and uneven group distribution, the ROC curve, random forest, and decision tree for DR detection may have been deficient. In addition, there was no detailed classification of systemic diseases or risk factors, and some indexes, such as diabetic foot, may have been undervalued due to the small number of subjects. In the future, more factors and more detailed classifications, such as IRF and HBP classifications, may be included to make the model more complete and thereby lower the misdiagnosis rate.

### Conclusion

The DR risk assessment protocol using the RETeval can be used for DR screening, but there is a relatively high missed diagnosis rate in the early stages of DR. In this study, FERG combined with the decision tree model was innovatively used to evaluate the risk of DR and improve the sensitivity of the protocol, which would be more suitable for DR screening.

## Data Availability Statement

The raw data supporting the conclusions of this article will be made available by the authors, without undue reservation.

## Ethics Statement

The studies involving human participants were reviewed and approved by the research ethics committee of Sun Yat-sen Memorial Hospital, Sun Yat-sen University. The patients/participants provided their written informed consent to participate in this study.

## Author Contributions

XD, ZL, and PZ have contributed equally to the work. All authors contributed to the article and approved the submitted version.

## Funding

Supported by the Natural Science Foundation of Guangdong Province (No.2015A030313019) and the Sun Yat-sen Clinical Research Cultivation Project (No.SYS-C-201705).

## Conflict of Interest

The authors declare that the research was conducted in the absence of any commercial or financial relationships that could be construed as a potential conflict of interest.
